# Intervention research in health promotion: policy issues, the example of the ten-year cancer strategy in France

**DOI:** 10.1093/eurpub/ckac129.073

**Published:** 2022-10-25

**Authors:** J Foucaud

**Affiliations:** Research Department, French National Cancer Institute, Boulogne-Billancourt, France

## Abstract

Prevention research covers a wide domain of studies, including identification of unknown risk factors for certain cancers, for example prostate cancer; or health promotion with approaches by population, by living environment or by territory. This research focuses in particular on identified carcinogenic factors (e.g. tobacco, alcohol, certain pesticides) but also on the causes of the prevalence of these factors in certain populations. PHIR makes it possible to meet these challenges. In 2010, the French National Cancer Institute was a pioneer in the development of PHIR in France and decided in 2021 to make it one of the spearheads of its ten-year strategy. This presentation will aim to give an account of this policy of developing RISP as a relevant tool for thinking about health promotion policies and actions, by targeting the issue of inequalities as one of the priority research objects as well as certain audiences (young people, vulnerable populations). The presentation will discuss four challenges for the implementation of this research policy: the development of the French scientific community in order to be able to respond to priority issues; the structuring and animation of this community; the evaluation of research projects and the mobilisation of a still timid global community; the articulation of academic and concrete/experiential knowledge. Through a concrete example of a national research development policy, the aim of this presentation is to help participants understand the challenges of RISP (its obstacles and levers, not limited to financial issues), the organisation of research, and the links between the actors involved (researchers and field workers, beneficiaries, health professionals). The first part will focus on identifying the participants’ representations of the obstacles to the development of RISP. The second part will be an interactive presentation of 12 minutes, the last 2-3 minutes will be devoted to final questions.

